# Distribution of culture-positive microorganisms varies with severity of liver disease in patients hospitalized with SBP

**DOI:** 10.1097/HC9.0000000000000655

**Published:** 2025-02-03

**Authors:** Nadim Mahmud, Natalia Salinas Parra, Aaron L. Hecht, David E. Kaplan, Marina Serper

**Affiliations:** 1 Department of Medicine, Corporal Michael J. Crescenz VA Medical Center, Philadelphia, Pennsylvania, USA; 2 Department of Medicine, Division of Gastroenterology and Hepatology, Perelman School of Medicine, University of Pennsylvania, Philadelphia, Pennsylvania, USA; 3 Leonard Davis Institute of Health Economics, University of Pennsylvania, Philadelphia, Pennsylvania, USA; 4 Department of Biostatistics, Epidemiology & Informatics, Center for Clinical Epidemiology and Biostatistics, Perelman School of Medicine, University of Pennsylvania, Philadelphia, Pennsylvania, USA; 5 Department of Medicine, Perelman School of Medicine, University of Pennsylvania, Philadelphia, Pennsylvania, USA

**Keywords:** bacterial translocation, Child-Turcotte-Pugh, cirrhosis, culture data, Veterans Health Administration

## INTRODUCTION

Spontaneous bacterial peritonitis (SBP) is a common complication of decompensated cirrhosis with high mortality, and patients may have evidence of concomitant infection in other sources, including the bloodstream and urinary tract.^1^ The most common isolates from ascitic fluid are gram-negative enteric organisms (primarily *Escherichia coli* and *Klebsiella*) due to gut bacterial translocation^2^,^3^; however, the microorganism profile of culture data in other sources has not been well studied. Delineating the epidemiology of SBP across infectious sources and patient characteristics is important in identifying high-risk patients, tailoring effective antibiotic regimens, and antibiotic stewardship. We aimed to (1) investigate the distribution of organisms among patients with positive cultures (peritoneal fluid, blood, and urine) in a national cohort of patients with cirrhosis hospitalized with SBP and (2) model the risk of 30-day mortality in patients with positive versus negative cultures.

## METHODS

This was a retrospective cohort study of adult patients with cirrhosis in the Veterans Outcomes and Costs Associated With Liver Diseases cohort^4^,^5^,^6^ with index hospitalization for SBP between January 2008 and October 2023. SBP was defined by paracentesis-obtained ascites fluid polymorphonuclear leukocyte count >250/mm^3^ in the hospital or up to 7 days prior to hospitalization as per prior methods.^7^ Baseline demographic data, body mass index, etiology of cirrhosis, MELD-Na prior to hospitalization, and MELD-Na on hospital presentation were collected. We analyzed culture growth stratified by source (blood, peritoneal, and urine samples) and Child-Pugh-Turcotte (CTP) class. In preparation for modeling of 30-day mortality, culture sources and results were categorized as urine/blood/peritoneal culture−; isolated urine culture+; blood culture+/peritoneal culture−, blood culture−/peritoneal culture+; and blood culture+/peritoneal culture+. A logistic regression model was fit with this primary exposure adjusted for age, sex, body mass index, race, etiology, prehospital MELD-Na, and diabetes (Supplemental Methods, http://links.lww.com/HC9/B891). Data management and statistical analyses were performed using STATA/BE 18.0 (College Station, TX).

## RESULTS

**FIGURE 1:**
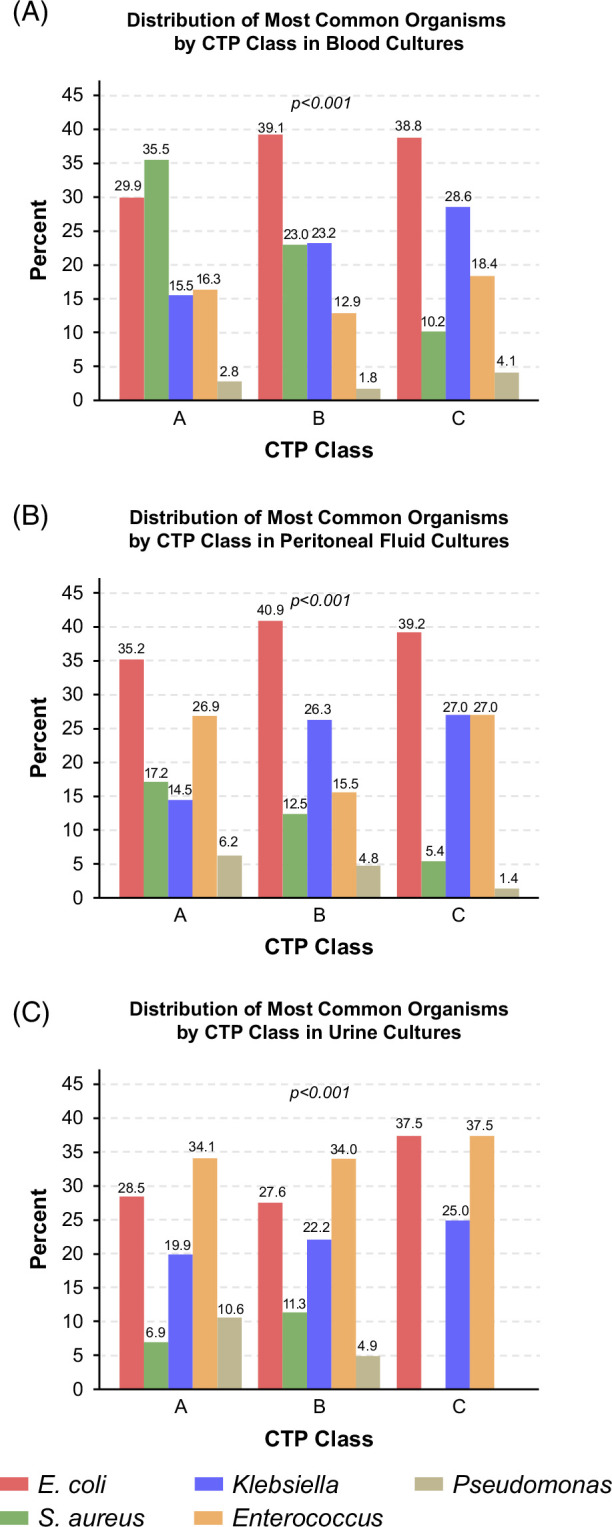
Distribution of most common organisms by CTP class in (A) blood cultures (B) peritoneal fluid cultures, and (C) urine cultures. Abbreviation: CTP, Child-Pugh-Turcotte.

A total of 5176 patients were included from 125 Veterans Health Administration centers; SBP was diagnosed at median hospital day 1 (IQR 0, 3). Patients were predominantly male (98%) and White (62%) with alcohol-associated liver disease (43.7%). On average, there was an increase in median MELD-Na from 16 to 24 at the time of hospitalization (Supplemental Table S1, http://links.lww.com/HC9/B891). Culture data identified organisms in 1748 patients (33.8%), with the majority identified in peritoneal fluid followed by blood and urine. *E. coli* was the most common organism across all fluid sources (urine 21.2%, blood 22.3%, peritoneal fluid 24.3%; *p*<0.001). There were significant differences in organism distribution across CTP classes for blood, peritoneal, and urine cultures (each *p*<0.001; Figure 1A–C). *E. coli* prevalence increased with liver disease severity in these groups (CTP A 29.9% vs. C 38.8% in blood; 35.2% vs. 39.2% in peritoneal fluid; 28.5% vs. 37.5% in urine), as did *Klebsiella* (eg, CTP A 15.5% vs. C 28.6% in blood). By contrast, *S. aureus* prevalence declined with increasing severity of liver disease (CTP A 35.5% vs. C 10.2% in blood; 17.2% vs. 5.4% in peritoneal fluid; 6.9% vs. 0.0% in urine). *Enterococcus* species were highly prevalent in urine cultures across all CTP classes (range 34.0%–37.5%).

In adjusted analysis, patients who were blood culture+/peritoneal culture+ had a 2.49-fold increased odds of 30-day mortality versus culture− patients (95% CI: 1.92–3.22; Supplemental Table S2, http://links.lww.com/HC9/B891). Patients who were blood culture−/peritoneal culture+ had 1.47-fold increased odds of 30-day mortality versus those who were culture− (95% CI: 1.23–1.77), and patients who were blood culture+/peritoneal culture− had a 1.38-fold increased odds of mortality versus culture− patients (95% CI: 1.12–1.66); all *p*<0.001.

## DISCUSSION

In this large cohort, there were significant differences in the microbiology of organisms in the blood, peritoneal fluid, and urine of patients with SBP across CTP classes. Higher CTP class was associated with higher rates of *E. coli* and *Klebsiella* infections and less *S. aureus* across all culture sources. The observed differences may reflect microbiome changes and potential differences in susceptibility to infection by selected organisms. Additionally, medication effects may impact the risk of selected microorganism infections. Lactulose and rifaximin, which are used for the treatment of HE, are known to alter the microbiome and increase the colonization of organisms in the gut.^8^ Prior studies also report that antibiotic overuse, nosocomial infections, and an increase in invasive procedures may shift the infectious landscape of SBP.^3^,^9^,^10^ However, no prior studies have explored the changing epidemiology of microorganisms across liver disease severity and multiple culture data sources. Further studies are needed to build on our findings and investigate the microorganism profile of patients treated for SBP and other infections and to evaluate resistance patterns based on specific antimicrobial exposures.

Our study also demonstrated higher odds of 30-day mortality in patients with positive cultures from both blood and peritoneal fluid, followed by positivity in one source or the other, relative to culture-negative patients. It is likely that multiple positive cultures indicate a more disseminated infection in an already vulnerable host with decompensated cirrhosis. This finding provides insight into patient prognosis and supports early identification and aggressive interventions with appropriate antimicrobial therapy for these higher-risk patients. Consistent with our findings, concurrent bacteremia has been associated with poor outcomes in SBP resulting in higher mortality.^2^ Finally, urine culture positivity did not impact the odds of mortality; asymptomatic bacteriuria is possible in many cases, though similar rates of *E. coli* and *Klebsiella* in urine cultures suggest a shared underlying source (ie, gut translocation).

Several limitations exist in this study. First, there are external validity limitations given that the Veterans cohort is predominantly male, largely White, and skewed toward HCV and alcohol-associated liver disease. Second, given the retrospective nature of this study, residual confounding is possible. Third, we were not able to explore antibiotic resistance patterns in this cohort or the influence of prior antibiotic exposure on microorganism distributions. These mark important areas of future inquiry.

In conclusion, we observed changing distributions of microorganisms from culture data across CTP classes in patients hospitalized with SBP. Patients with positive culture data in both blood and peritoneal cultures had the highest risk of 30-day mortality, underscoring the need for early detection and aggressive management in this high-risk population.

## Supplementary Material

SUPPLEMENTARY MATERIAL
