# Supplementary material for: Distribution of culture-positive microorganisms varies with severity of liver disease in patients hospitalized with SBP
Source: Hepatol Commun. 2025 Feb 3;9(2):e0655. doi: 10.1097/HC9.0000000000000655 (PMC12333798; doi:10.1097/HC9.0000000000000655)
Supplement: SUPPLEMENTARY MATERIAL [file hc9-9-e0655-s001.docx]

**Supplemental Methods**

*Construction of Multivariable Logistic Regression Model*

To fit an adjusted model for 30-day mortality with a primary exposure of culture source and result, we considered adjustment for all pre-hospital exposures noted in **Supplemental Table 1**. The rationale for this was to avoid adjustment for mediators between infection and the outcome. Reverse stepwise variable selection was used to select a candidate model, after which multiple modified clinician-driven models were evaluated where variables felt to be clinically meaningful were reintroduced, or less clinically salient variables removed. All candidate models were then compared in terms of Aikake and Bayesian Information Criteria (AIC and BIC), with final model selection based on minimization of these criteria.

**Supplemental Table 1 – Cohort Characteristics**

|  | **Overall Cohort** | **Stratified by Pre-Hospitalization CTP Class** | | | |
| --- | --- | --- | --- | --- | --- |
| **Factor** | **Value (N=5,176)** | **CTP A (N=2,388)** | **CTP B (N=2,421)** | **CTP C (N=306)** | **p-value** |
| Age, median (IQR) | 62 (57, 67) | 64 (59, 69) | 61 (56, 66) | 58 (53, 63) | <0.001 |
| Male Sex | 5070 (98.0%) | 2,345 (98.2%) | 2,371 (97.9%) | 294 (96.1%) | 0.048 |
| BMI, median (IQR) | 27.5 (24.0, 31.5) | 27.1 (23.5, 31.3) | 27.7 (24.3, 31.7) | 27.8 (24.6, 31.5) | <0.001 |
| Race/Ethnicity |  |  |  |  | 0.019 |
| White | 3209 (62.0%) | 1,479 (61.9%) | 1,490 (61.5%) | 198 (64.7%) |  |
| Black | 727 (14.0%) | 377 (15.8%) | 312 (12.9%) | 31 (10.1%) |  |
| Hispanic | 495 (9.6%) | 217 (9.1%) | 242 (10.0%) | 29 (9.5%) |  |
| Asian | 58 (1.1%) | 25 (1.0%) | 30 (1.2%) | 2 (0.7%) |  |
| Other | 687 (13.3%) | 290 (12.1%) | 347 (14.3%) | 46 (15.0%) |  |
| AUDIT-C Score, median (IQR) | 0 (0, 3) | 0 (0, 3) | 0 (0, 3.75) | 0 (0, 4) | 0.29 |
| Etiology of Liver Disease |  |  |  |  | <0.001 |
| Hepatitis C Virus (HCV) | 555 (10.7%) | 284 (11.9%) | 239 (9.9%) | 25 (8.2%) |  |
| Hepatitis B Virus | 33 (0.6%) | 17 (0.7%) | 14 (0.6%) | 2 (0.7%) |  |
| Alcohol-related Liver Disease (ALD) | 2261 (43.7%) | 985 (41.2%) | 1,101 (45.5%) | 150 (49.0%) |  |
| HCV + ALD | 1430 (27.6%) | 621 (26.0%) | 699 (28.9%) | 100 (32.7%) |  |
| MASLD | 780 (15.1%) | 420 (17.6%) | 318 (13.1%) | 24 (7.8%) |  |
| Other | 117 (2.3%) | 61 (2.6%) | 50 (2.1%) | 5 (1.6%) |  |
| MELD-Na Pre-Hospital, median (IQR) | 16 (11, 22) | 12 (8, 17) | 20 (15, 24) | 26 (22, 30) | <0.001 |
| Coronary Artery Disease | 1271 (24.6%) | 654 (27.4%) | 538 (22.2%) | 62 (20.3%) | <0.001 |
| Diabetes Mellitus | 3036 (58.7%) | 1,468 (61.5%) | 1,344 (55.5%) | 186 (60.8%) | <0.001 |
| Atrial Fibrillation | 605 (11.7%) | 286 (12.0%) | 284 (11.7%) | 25 (8.2%) | 0.14 |
| Loop Diuretics Pre-Hospital | 3087 (59.6%) | 1,135 (47.5%) | 1,677 (69.3%) | 269 (87.9%) | <0.001 |
| Potassium-Sparing Diuretics Pre-Hospital | 2701 (52.2%) | 871 (36.5%) | 1,560 (64.4%) | 267 (87.3%) | <0.001 |
| Hepatic Encephalopathy Medications Pre-Hospital | 1817 (35.1%) | 523 (21.9%) | 1,065 (44.0%) | 228 (74.5%) | <0.001 |
| Non-Selective Beta Blockers Pre-Hospital | 1780 (34.4%) | 659 (27.6%) | 958 (39.6%) | 160 (52.3%) | <0.001 |
| MELD-Na at Hospitalization, median (IQR) | 24 (19, 30) | 21 (16, 26) | 26 (21, 31) | 30 (25, 35) | <0.001 |
| Absolute Ascitic PMN Count, median (IQR) | 1104 (458, 3704) | 963 (427, 3,354) | 1,196 (477, 3,906) | 1,529 (490, 4,133) | <0.001 |
| Any Organism Identified on Cultures | 1748 (33.8%) | 709 (29.7%) | 884 (36.5%) | 135 (44.1%) | <0.001 |
| Urine Culture Positive | 484 (9.4%) | 226 (9.5%) | 211 (8.7%) | 37 (12.1%) | 0.14 |
| Blood Culture Positive | 831 (16.1%) | 297 (12.4%) | 461 (19.0%) | 63 (20.6%) | <0.001 |
| Peritoneal Culture Positive | 910 (17.6%) | 338 (14.2%) | 487 (20.1%) | 76 (24.8%) | <0.001 |

**Supplemental Table 2 – Multivariable Logistic Regression Model for 30-Day Mortality**

| **Variable** | **OR (95% CI)** | **p-value** |
| --- | --- | --- |
|  |  |  |
| **Culture Source and Result** |  |  |
| Urine, blood, peritoneal cultures negative | (reference) | - |
| ***Urine+ alone*** | 0.91 (0.68, 1.21) | 0.51 |
| ***Blood+***, Peritoneal-, Urine +/- | 1.36 (1.12, 1.66) | 0.002 |
| Blood-, ***Peritoneal+*,** Urine +/- | 1.47 (1.23, 1.77) | <0.001 |
| ***Blood+***, ***Peritoneal+***, Urine +/- | 2.49 (1.92, 3.22) | <0.001 |
| **Age** | 1.02 (1.01, 1.03) | <0.001 |
| **BMI** | 0.99 (0.98, 1.00) | 0.19 |
| **Etiology** |  |  |
| Hepatitis C Virus (HCV) | (reference) | - |
| Hepatitis B Virus | 0.70 (0.31, 1.55) | 0.38 |
| Alcohol-related Liver Disease (ALD) | 0.70 (0.57, 0.86) | 0.001 |
| HCV + ALD | 0.78 (0.63, 0.97) | 0.02 |
| MASLD | 0.66 (0.51, 0.85) | 0.001 |
| Other | 0.45 (0.28, 0.74) | 0.002 |
| **MELD-Na Pre-Hospital** | 1.02 (1.01, 1.03) | <0.001 |
| **Hepatic Encephalopathy Medications Pre-Hospital** | 1.35 (1.17, 1.55) | <0.001 |
| **Loop Diuretics Pre-Hospital** | 0.92 (0.80, 1.06) | 0.24 |
| **Diabetes Mellitus** | 0.74 (0.65, 0.84) | <0.001 |
